# Quantitative estimation of intravoxel incoherent motion parameters in acute ischemic stroke: A Systematic review and meta-analysis

**DOI:** 10.1186/s12880-025-01997-3

**Published:** 2025-11-12

**Authors:** Tancia Pires, Priyanka, Rajagopal Kadavigere, John M Solomon, Saikiran Pendem, Priya P. S, Nikith A. Shetty, Abhijith S.

**Affiliations:** 1 Department of Medical Imaging Technology, Manipal College of Health Professions, Manipal Academy of Higher Education, Karnataka, Manipal 576104 India; 2 Department of Radiodiagnosis and Imaging, Kasturba Medical College, Manipal Academy of Higher Education, Karnataka, Manipal 576104 India; 3 Present Address: Department of Physiotherapy, Manipal College of Health Professions, Manipal Academy of Higher Education, Karnataka, Manipal, 576104, India; Centre for Comprehensive Stroke Rehabilitation and Research, Manipal Academy of Higher Education, Manipal, Karnataka 576104 India; 4 Department of Neurology, Kasturba Medical College, Manipal Academy of Higher Education, Karnataka, Manipal 576104 India

**Keywords:** IVIM, Intravoxel incoherent motion, Acute ischemic stroke, Quantitative imaging, Diffusion-perfusion MRI

## Abstract

**Background:**

Intravoxel incoherent motion (IVIM) is a potential diagnostic technique for acute ischemic stroke as it simultaneously quantifies the molecular diffusion and the minute capillary network microcirculation; however, the lack of comprehensive evidence of its utility in acute ischemic stroke necessitates a review to enable better quantitative estimation and understanding of IVIM parameters and their relation with parameters of conventional diffusion and perfusion techniques.

**Methods:**

An extensive search strategy yielded 2340 studies in six databases. After a two-step screening process, data from 10 studies were extracted and included in the meta-analysis. Heterogeneity, publication bias, and forest plot analysis were performed for each IVIM parameter: D, D*, f, and fD*.

**Results:**

The mean values for D ranged from 0.28 to 0.58 × 10^− 3^ mm^2^/s and from 0.56 to 0.93 × 10^− 3^ mm^2^/s in the lesion and contralateral normal region, respectively, with the standardised mean difference of 3.24 SD units and positive correlation with ADC. f-values were between 2.5 and 4.9% ipsilaterally and 4.3 to 7.9% contralaterally, with minimal heterogeneity and a statistically significant standardised mean difference of 2.65 SD units. D* showed a non-significant effect. fD* showed a significant standardised mean difference of 1.48 SD units.

**Conclusion:**

Quantitative differentiation between the core and normal regions was significantly seen in D, f, and fD* parametric maps. Although D* varied across studies, technical upgrading could improve its utility. Since IVIM parameters correlate well with the existing diffusion and perfusion parameters, its utility offers a robust, multifaceted approach to understanding acute ischemic stroke.

**Clinical trial number:**

Not applicable.

**Supplementary Information:**

The online version contains supplementary material available at 10.1186/s12880-025-01997-3.

## Background

Globally, over 62% of all incident strokes are ischemic strokes. There are over 7.6 million new ischemic strokes each year [1]. Diffusion-weighted magnetic resonance imaging (DW-MRI) has been known to delineate the ischemic core and is accurate in identifying stroke mimics with great sensitivity. Perfusion-weighted imaging (PWI) sheds light on the viable perfused tissue in acute ischemic infarcts. A mismatch between these regions, known as the diffusion-perfusion mismatch, is used to approximate the penumbra and guide reperfusion therapy. Similarly, in CT perfusion, a mismatch between cerebral blood volume (CBV) and cerebral blood flow (CBF) provides a comparable estimate. However, these approaches require separate imaging sequences or modalities. PWI techniques currently available for acute ischemic stroke include dynamic susceptibility contrast (DSC), which involves susceptibility-sensitive imaging via the administration of an exogenous gadolinium-based contrast agent (GBCA), and arterial spin labelling (ASL), which involves the tagging of arterial blood and imaging the difference in magnetization of the tagged and untagged spins. Though this technique is non-invasive, compared to DSC, the parameters can be affected by magnetic inhomogeneities, low image quality, or large vessel pulsations [2, 3]. Moreover, GBCA used in DSC is contraindicated in pregnancy and in patients with defective kidney function as it could aggravate these issues, leading to nephrogenic systemic fibrosis (NSF) [4]. To reveal in-depth lesion characteristics in acute ischemic stroke, diffusion weighted imaging (DWI), as well as PWI sequences, must be evaluated. However, the time taken to perform these MRI sequences is a limitation in the acute phase of stroke, as around 1.9 million neurons are destroyed every minute [5]; hence, assessing the diffusion-perfusion mismatch within a single Intravoxel incoherent motion (IVIM) framework could simplify imaging protocols and reduce scan time, particularly in patients contraindicated for contrast-based studies [6].

IVIM is an MRI technique that quantifies the molecular diffusion and the minute capillary network microcirculation of water. It can provide diffusion and perfusion-related information separately in just one sequence using several b-values and a bi-exponential fitting curve [7]. The most common parameters assessed in this technique are the diffusion parameter D and perfusion-related parameters D*, f, and fD*. D is the true diffusion coefficient, which gives information about the thermal diffusion or brownian motion of water molecules in the tissue. D* is the pseudo diffusion coefficient that quantifies the motion of water molecules in the blood. Since proton transport by vascular flow is considerably faster than thermal diffusion, D* is also referred to as fast ADC (Apparent Diffusion Coefficient), while D as slow ADC; f – the perfusion fraction that reveals the fraction of pseudo-diffusion to the signal intensity in each voxel; and fD* is the product of two parameters f and D* related to the blood flow [8, 9]. A brief description of the IVIM parameters is tabulated in Table 1.

**Table 1:** Brief description of the IVIM parameters

Parameter		Description
D	True diffusion coefficient	Thermal diffusion or Brownian motion of water molecules in the tissue
f	Perfusion fraction	Fraction of pseudo-diffusion to the signal intensity in each voxel
D*	Pseudo diffusion coefficient	Quantifies the motion of water molecules in the blood
fD*	Related to CBF	Product of two parameters f and D* related to the blood flow

Numerous studies have assessed the utility of IVIM in diagnosing tumors in organs like the brain, breasts, kidneys, salivary glands, pancreas, or liver; it has also been tested for differentiating benign and malignant tumors or low-grade from high-grade tumors [10–15]. Given the high burden of disability and the narrow therapeutic window in acute ischemic stroke, there is an urgent clinical need for imaging techniques that can rapidly and safely assess tissue viability. IVIM MRI, by providing simultaneous diffusion and perfusion-related information without contrast agents, holds potential for identifying the infarct core and penumbra, thereby informing timely therapeutic interventions. It also extends the information provided by ADC by separating diffusion (D) and perfusion (D*, f) components within the same acquisition, while ADC reflects a composite signal influenced by both true molecular diffusion and microcirculation, IVIM enables compartmentalized analysis, which may be especially beneficial in ischemic stroke where both diffusion restriction and perfusion deficits are central to pathology [16].

Studies have also been performed on acute stroke, and the parameters obtained using IVIM in the ischemic core on the ipsilateral side and the contralateral normal region of the brain have shown its potential as an effective tool for acute stroke assessment [17]. However, only a few studies have been performed, some with comparatively smaller sample sizes [18–21]; while others had dynamic ranges and variations among parameters [17, 22, 23]. Also, the first study performed in acute ischemic stroke patients using IVIM showed a decrease in IVIM parameters in the core as compared to the control in the acute phase [24]. In contrast, studies focusing on subacute or chronic infarcts have shown an increase in the diffusion parameter (D), emphasizing the need to better understand the temporal evolution of IVIM parameters. This highlights the importance of cross-study comparisons worldwide to more accurately characterize parameter behavior, particularly during the acute phase, and enhance the differentiation between infarct stages. Lately, due to upgrades in hardware and software and ongoing research, this technique has been gaining popularity. Nonetheless, to our knowledge, no compilation of IVIM parametric findings is available so far that provides insight into the degree of variation of parameters or reference values in the ischemic lesion or the normal brain tissues. Nor does the information regarding the relation of IVIM parameters in acute ischemic stroke with the conventional diffusion and perfusion techniques exist. Hence, this systematic review and meta-analysis aims to identify the range of values of the various IVIM parameters in the ischemic lesion and the contralateral normal hemisphere of the brain and assess whether the pooled effect across studies in these parameters is significant. Also, the relationship between IVIM parameters and DSC or ASL parameters, if any, will be highlighted, thus enabling a better understanding of IVIM parameters and ensuring consistency in diagnosing acute ischemic stroke.

## Methodology

This review has been registered in PROSPERO (CRD42024558531) and is created following the PRISMA (Preferred Reporting Items for Systematic Reviews and Meta-Analyses) 2020 checklist (Supplementary Material S1) and guidelines [25].

### Search method and resources

Six databases, i.e., PubMed, Embase, Scopus, CINAHL Ultimate, Google Scholar, and Web of Science, were extensively and systematically searched using a search strategy with appropriate keywords and MeSH terms pertaining to IVIM and Stroke (Available as supplementary material S2). The search strategy was then adapted to each database appropriately. One reviewer (TP) searched the databases from its inception till June 2024. A hands-on search of grey literature and reference lists of retrieved full texts was also conducted to ensure coverage of extensive literature.

### Study selection process

All the articles were uploaded to the software for de-duplication and screening. Two independent reviewers (TP and PR) screened the title and abstract, followed by a full-text assessment. Any conflict was resolved by discussion with a third moderator (RK). The inclusion criteria were based on the PICOTS framework, where studies that performed IVIM in acute ischemic stroke and analyzed the variations among its parametric maps were included. The detailed inclusion and exclusion criteria are outlined in Table 2.

**Table 2:** Detailed criteria for inclusion and exclusion of studies

Inclusion Criteria	Exclusion Criteria
**Population**: Studies involving individuals having first-ever, hemispheric stroke with MRI-Brain performed in the acute phase	Conference abstracts, notes, book chapters, editorial letters, case reports, series, and reviews
**Intervention/Acquisition**: IVIM sequence performed, with at least 2 of the following parameters calculated: *D*, D*,* f*, and/or fD*	Studies performed on animal models of stroke.
**Comparison**: IVIM measurements from the ischemic lesion/core compared to the corresponding contralateral normal (control) region of interest (ROI).	Studies including patients with transient ischemic attacks
**Outcomes/Effects**: 1. Differentiation of lesioned and the contralateral regions by IVIM parameters. 2. Correlation between IVIM parameters and parameters obtained from other existing diffusion and perfusion techniques	Studies involving patients with any prior intervention (e.g. mechanical thrombolysis) that would affect the range of IVIM parameters
**Time frame**: Patients in the acute phase with symptom onset to MRI examination time of < 6 days	Studies unavailable as full text in English.
**Setting**: Hospital-based quantitative retrospective or prospective studies	Foreign language articles in non-translatable formats.

### Data extraction and risk of bias

Two reviewers independently extracted the following data:

*Study characteristics*: First author, year of publication, place and duration of the study, study design, and sample size.

*Patients’ demographic data*: age, sex, baseline NIHSS score, and symptom onset to imaging time.

*MRI data*: field strength and type of MRI machine used, number of b-values, IVIM protocol parameters, and post-processing technique used.

*Outcome measures*: the lesion and contralateral D, D*, f, fD*, ADC, and other ASL and DSC parameters, if available, like CBF, CBV, MTT (Mean Transit Time), etc.

Two independent reviewers assessed the quality of the included studies using the Joanna Briggs Institute (JBI) critical appraisal tool. Conflicts in the assessment were sorted out through discussion with a senior reviewer (JS).

### Statistical analysis

Meta-analysis was performed using the MAJOR module of Jamovi 2.3.28 software. Heterogeneity across studies was assessed using the I^2^ test. A random effects model was used if I^2^ > 50%; if not, a fixed effects model was used. The forest plots were generated using Cohen’s *d* as the effect size, using the formula *d* =​ M1​−M2/SD pooled, which represents the standardized mean difference (SMD) between the core and control groups (ipsilateral and contralateral mean value of the IVIM parameter, respectively). As Cohen’s *d* is a dimensionless measure expressed in standard deviation units, the x-axis of the plot reflects standardized differences rather than raw outcome units. Each study’s effect size was plotted with its 95% confidence interval, and the line of no effect was set at *d* = 0. For studies where the standard error (SE) or 95% confidence interval (CI) of the paired difference was not mentioned, the approximation method was used [26]. Publication bias was assessed using Fail-Safe N, Kendall’s Tau, and Egger’s regression tests. Sensitivity analyses were performed to assess the robustness of the pooled effect estimates. Studies with effect size values exceeding two standard deviations from the standardized mean difference were considered outliers and excluded from sensitivity analyses.

## Results

### Literature search results

A comprehensive search across six databases yielded 2,340 articles. Of these, 1,003 duplicates were removed, and 957 records were excluded as irrelevant. The remaining 380 records underwent title and abstract screening, during which 362 were excluded. Approximately half of these exclusions were studies evaluating the utility of IVIM in non-stroke populations (e.g., tumors, adnexal masses, liver lesions). Around 20% focused on subacute or chronic stroke patients or transient ischemic attacks, while another 20% comprised reviews, conference abstracts, editorials, or preclinical studies. The remaining 10% included studies that investigated diffusion or perfusion metrics using methods other than IVIM imaging, such as ADC mapping, Diffusion kurtosis imaging (DKI)etc.

**Fig. 1:**
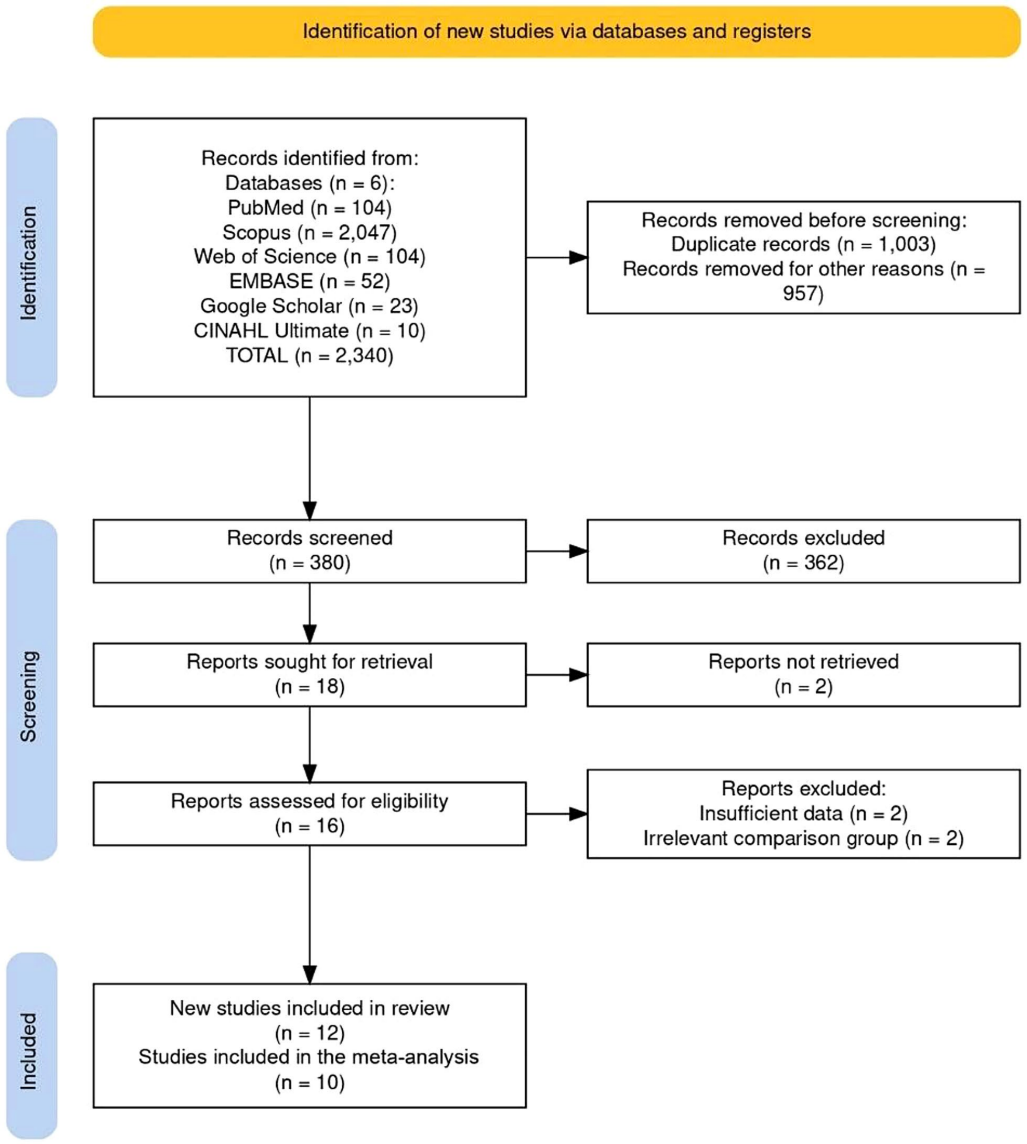
PRISMA flowchart of the study inclusion process

Ultimately, 18 studies proceeded to full-text assessment. Of these, two articles could not be retrieved, and four were excluded as the studies either reported only one IVIM parameter, lacked comparison with the contralateral hemisphere, or did not perform correlation analysis with other imaging modalities. The detailed reasons for exclusion are outlined in the flow diagram (Fig. 1). Finally, 12 studies [17–24, 27–30] were included for review and 10 for the meta-analysis [17–19, 21–23, 27–30].

### Characteristics of included studies

A total of 386 patients were recruited in the 12 studies included in the review. The mean age ranged from 47 to 75 years, with a pooled mean of 66.9 ± 14 years. Sex distribution across studies was approximately 225 males and 146 females. Six studies were prospective, while six were retrospective. Patients enrolled belonged to North America [23, 28], Europe [18, 21, 24, 27, 30], and Asia [17, 19, 20, 22, 29]. The baseline NIHSS scores were reported in most studies and ranged from 7 to 18. Most studies (9/12) used 3 Tesla (T) MRI scanners, while three used 1.5T scanners. Many of the studies used General Electric (GE) scanners, e.g., MR750 and HDxt. Siemens and Philips Achieva were among the other scanners used. Details are shown in Table 3.

**Table 3:** Study characteristics and patient demographics extracted from the studies included

Sr. no.	First author	Year	Place	Study direction	Sample size (*N*)	No. of Male/Female	Age mean ± SD	NIHSS Score mean ± SD	Additional parameters analysed.
1	Wirestam et al [24]	1997	Sweden	P	15	NR	47.5 ± 13.44	NR	NR
2	Federau et al [18]	2014	Switzerland and USA	P	17	12/5	56.2 ± 23.4	7.7 ± 5.4	NR
3	Suo et al [17]	2015	China	R	101	63/38	63.6 ± 12.9	7.13 ± 7.03	**ADC** with D (*r* = 1, *p* < 0.001) and f (*r* = 0.541, *p* < 0.001
4	Hu et al [20]	2015	China	R	15	11/4	68.7 ± 8.0	NR	D* with **ASL-CBF** (*r* = 0.416; *p* = 0.022)
5	Yao et al [19]	2016	China	P	38	26/12	55 (23–74)^#^	NR	fD* with **ASL-CBF** (*r* = 0.653, *p* < 0.001); f with **ASL-CBF** (*r* = 0.472, *p* = 0.012)
6	Federau et al [27]	2019	Switzerland	R	34	17/17	68.6 ± 14.3	15.5 ± 7.2	NR
7	Zhu et al [28]	2019	USA	R	20	11/9	66.0 ± 12.5	18.4 ± 7.5	fD* and **DSC-CBF** (Best agreement -Bland altman analysis)
8	Zhu et al [23]	2019	USA	R	58	36/22	70.2 ± 13.4	15.9 ± 6.7	NR
9	Chen et al [29]	2021	China	P	39	19/20	69 ± 13	13 ± 6	NR
10	Yamashita et al [22]	2022	Japan	R	29	17/12	75.2 ± 12	6.1 ± 7.4	NR
11	Pavilla et al [21]	2022	France	P	5	4/1	75 ± 11	14.2 ± 5.3	NR
12	Pavilla et al [30]	2023	France	P	15	9/6	71 ± 11	7.7 ± 5.5	**ADC** and D (*r* = 0.599, *p* < 0.0001)

P = Prospective, R = Retrospective, T*=Tesla, #given in the form Median (IQR), NR = Not Reported

### Risk of bias assessment

**Fig. 2:**
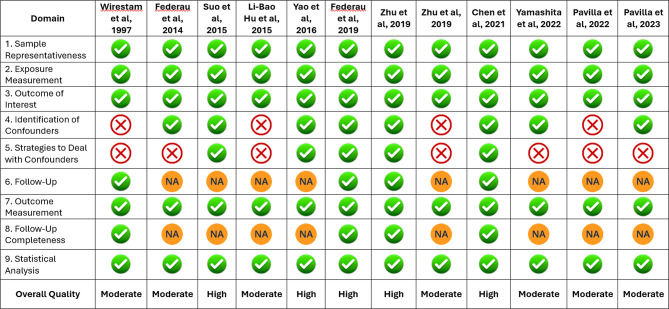
JBI Joanna Briggs Institute Critical appraisal chart

The risk of bias was assessed for all 12 studies included in the review using the JBI critical appraisal tool. This tool has 9 domains based on which the quality of a study is assessed. Seven out of 12 studies were of moderate quality, and five others were of high quality with a low risk of bias. The assessment revealed that most studies scored high in terms of sample representativeness, exposure and outcome measurement, and statistical analysis. However, several studies showed limitations in identifying and addressing confounding variables, particularly older studies and those with smaller sample sizes. A diagrammatic representation with elaborate detail is given in Fig. 2.

### The outcome measures

The outcome measures assessed for meta-analysis were the IVIM parameters D, f, D*, and fD* in both the lesion and the contralateral normal region, outlined in supplementary material Table S3. Since each parameter from the included studies had dynamic ranges and variations, the meta-analysis used the effect sizes and standard variances to determine meaningful observed differences. Further, the established correlation between IVIM parameters with parameters like ADC (DWI), CBF, CBV, and MTT from DSC or ASL from studies that performed such comparisons is enumerated.

Of the 12 studies listed in Table 2, two were excluded from meta-analysis: Wirestam et al. [24], which reported only a single IVIM parameter (f), and Hu et al. [20], which analyzed ROIs in the penumbra and normal tissue rather than the infarct core and its contralateral counterpart. These studies were therefore not suitable for pooled quantitative comparison and hence were excluded from the meta-analysis. The imaging-related variables extracted from the 10 included studies are tabulated in the supplementary material Table S4.

### D – true diffusion coefficient

**Fig. 3:**
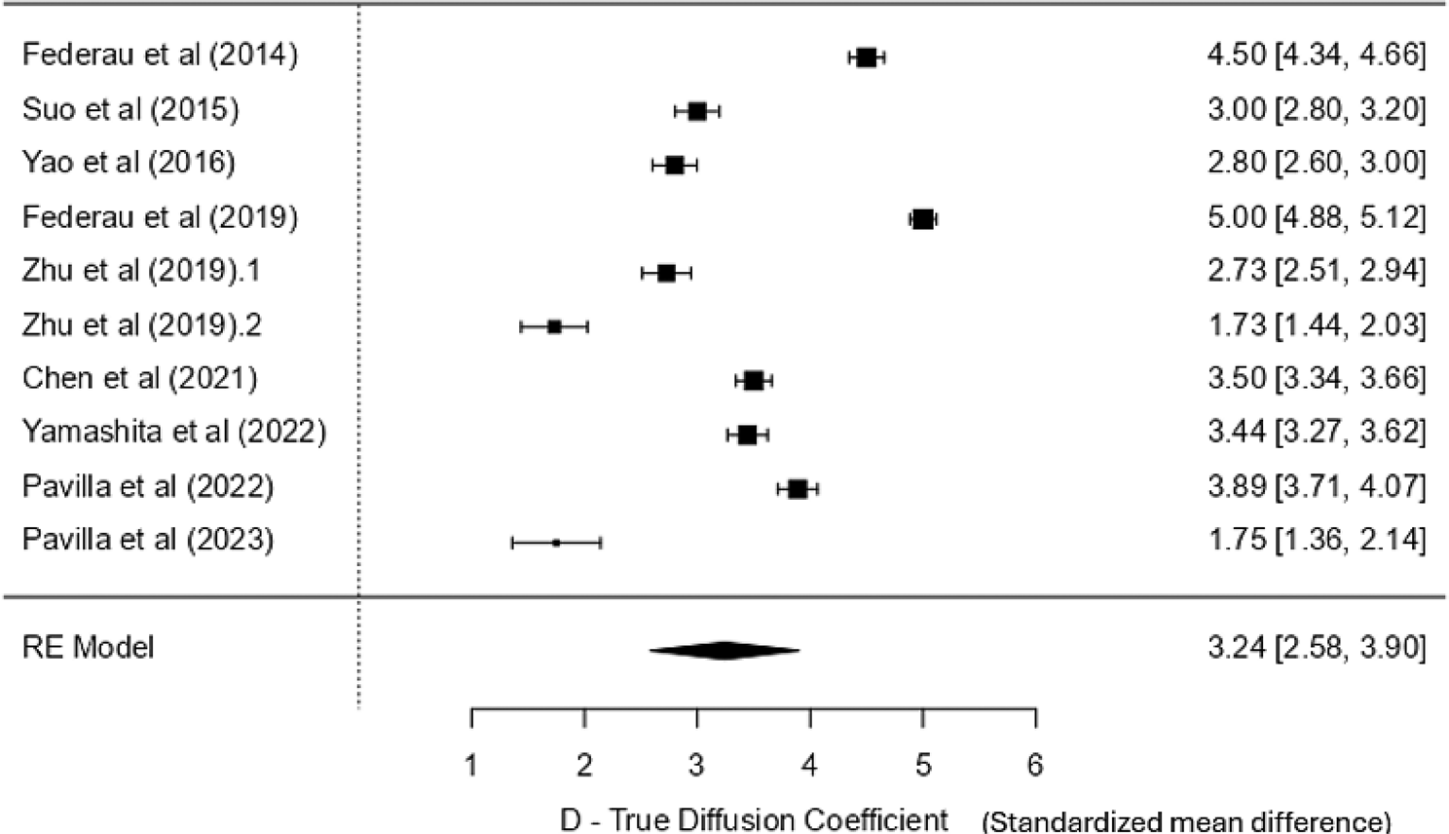
Forest plot for D -true diffusion coefficient

Considering all 10 studies, it was evident that the values in the lesion core on the ipsilateral side were significantly lower than those in the normal tissue in the contralateral hemisphere. The mean values for D in the lesion/core ranged from 0.28 to 0.58 × 10^− 3^ mm^2^/s. The weighted mean in the core is 0.439 × 10^− 3^ mm^2^/s (0.429–0.449 at 95% CI), whereas the contralateral normal tissue mean values ranged from 0.56 to 0.93 × 10^− 3^ mm^2^/s. The weighted mean (control) was 0.885 × 10^− 3^ mm^2^/s (0.880–0.889 at 95% CI). No particular trend was seen in the parametric values based on the MRI field strength and the number of b-values used. A high heterogeneity (I^2^ = 99%) indicates that nearly all observed variance is due to heterogeneity rather than sampling error. Hence, a random effect model was used for the analysis. The standardised mean difference (SMD) was 3.24 SD units (95% CI: 2.58–3.90) (Fig. 3), which was statistically significant (*p* < 0.001), indicating that the true diffusion coefficient (D) differs significantly from zero and enables the clinical differentiation between the core and the normal tissue. The Z-value (9.65) also supports the strong significance of the result. Although there is some indication of publication bias (significant Kendall’s Tau − 0.828 and Egger’s tests − 4.974), the large Fail-Safe N (50,021) suggests that the overall conclusion is robust despite the potential bias.

### f – the perfusion fraction

**Fig. 4:**
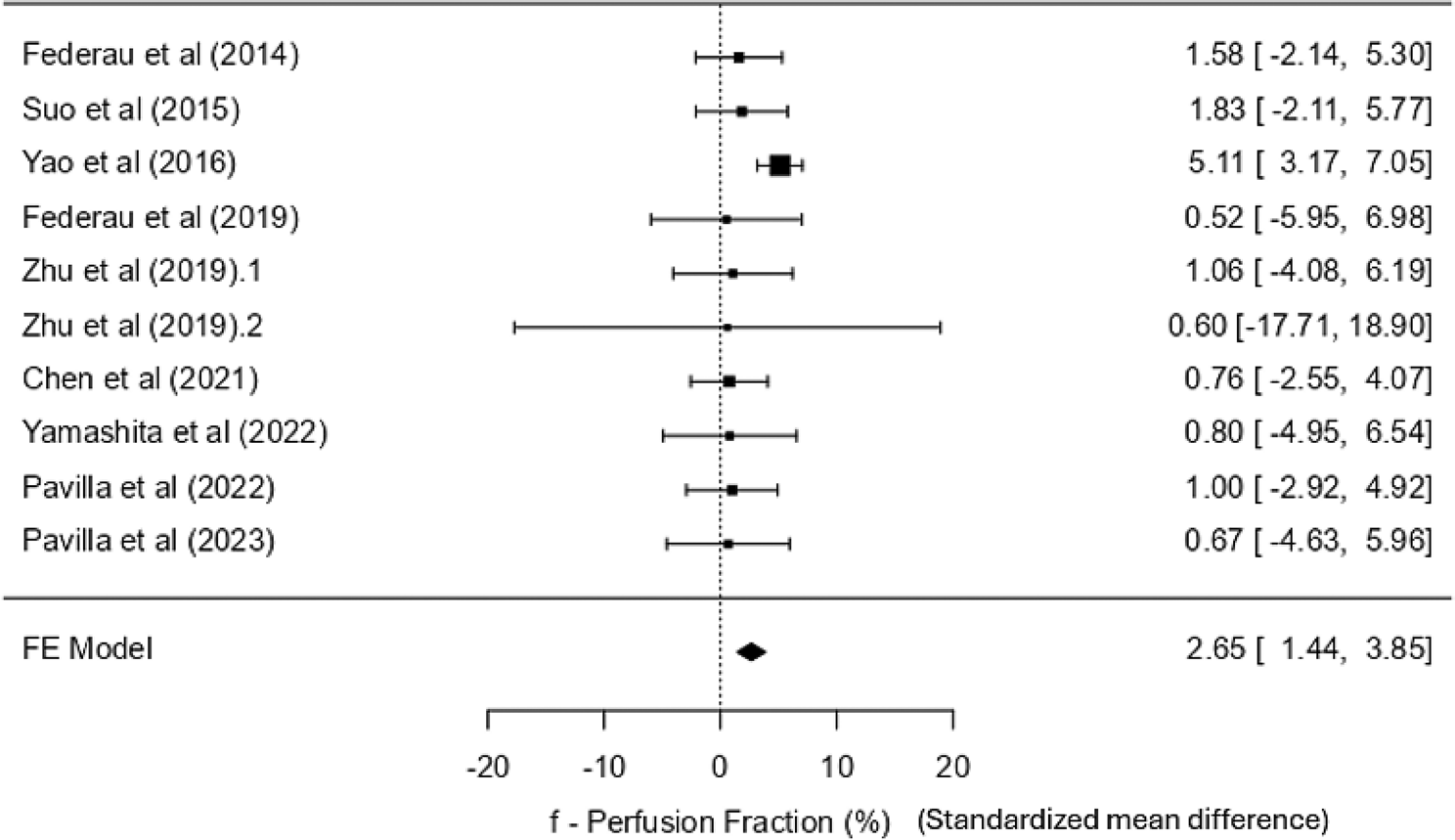
Forest plot for f -perfusion fraction

All ten studies reported the f (perfusion fraction) statistic, both in the core and the contralateral normal tissue. Except for extreme values (core: 16.63 ± 9.34%; control: 22.19 ± 6.53%) from a study by Zhu et al. [23] The values in the core ranged from 2.5 to 4.9%, and in the contralateral normal tissue, 4.3 to 7.9%. The weighted mean of *f* in the core was 3.661% (3.461, 3.862 at 95% CI); while it was 7.007% (6.793, 7.222 at 95% CI) in the control. The heterogeneity statistics suggest minimal variability between studies. The low I² (13.26%) supports the appropriateness of using a fixed-effects model that yielded an SMD of 2.65 SD units (SE = 0.615), indicating a statistically significant effect (*p* < 0.001) with a low SE, suggesting consistency across studies, thereby enabling the differentiation between the core and control regions based on the perfusion properties of the tissue. The 95% confidence interval for the SD mean difference ranged from 1.444 to 3.853, and the Z-value of 4.31 further confirms the robustness of this finding. There is some evidence of publication bias (significant Egger’s test − 2.366, *p* = 0.018). A negative value suggests that smaller studies tend to report larger effect sizes. However, the other tests (Fail-Safe *N* = 20 and Kendall’s Tau = 0.022) suggest minimal bias. The forest plot (Fig. 4) shows that the confidence interval of most individual studies crosses the line of no significance; however, the summary effect shows a significant difference of almost 26%.

During sensitivity analyses, the study by Zhu et al. [23] was identified as an outlier, as its effect size values were more than two standard deviations from the standardised mean difference, and was therefore excluded according to our predefined criterion. After this exclusion, a slightly adjusted standardised mean difference of 2.66 (95% CI: 1.451 to 3.865) that remains statistically significant (*p* < 0.001) was seen. Since the results remained consistent, the forest plot for sensitivity analysis has not been included.

### D* - pseudo diffusion coefficient

**Fig. 5:**
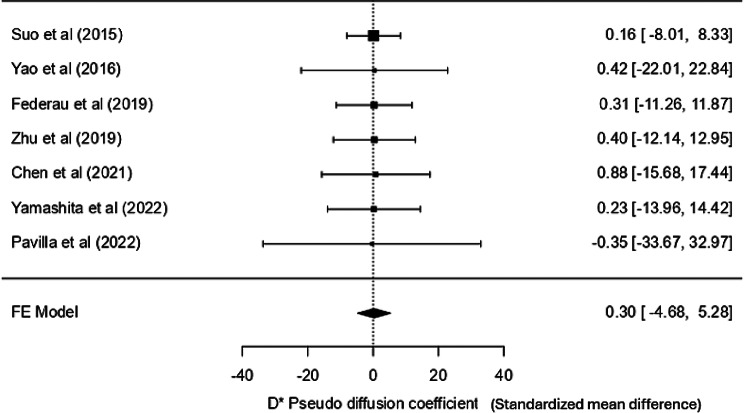
Forest plot for D* -pseudo-diffusion coefficient sensitivity analysis

Out of 10, only eight studies mentioned the D* statistic. Most studies showed that the values in the lesion were lower than the contralateral normal tissue, except one [21]. However, the values varied drastically from one study to another, with overlapping values in the core and the control regions, so using a specific cut-off value or a range is not feasible. Furthermore, the weighted mean in the core was 13.223 × 10^− 3^ mm²/s (12.558, 13.888 at 95% CI) and 19.095 × 10^− 3^ mm²/s (18.431, 19.760 at 95% CI) in the control. Moreover, the fixed-effects model analysis yielded a non-significant SMD of 0.301 (SE = 2.54, Z = 0.118, *p* = 0.906), with a CI ranging from − 4.680 to 5.282, indicating no significant effect. Heterogeneity among the studies was negligible (I² = 0%, Q = 0.008, *p* = 1.000), so variability in effect sizes was minimal. Publication bias assessments, including Fail-Safe N, Kendall’s Tau, and Egger’s regression tests, all indicated no significant bias. This meta-analysis confirms the lack of a significant effect for the D* pseudo diffusion coefficient. The study by Zhu et al. [23], with an exceptionally large confidence interval, was removed in a sensitivity analysis to improve visual interpretation. However, the pooled effect remained statistically insignificant (Fig. 5). The forest plot, including all studies, is provided in the supplementary material S5 (Fig. S5a).

### fD* - statistic related to blood flow (product of perfusion fraction and pseudo diffusion coefficient)

Excluding the unusually large values (Core: 520.3 ± 631.2 × 10^− 3^ mm²/s; Control: 752.4 ± 545.8 × 10^− 3^ mm²/s) from one study [23], the fD* values in the core ranged from 0.16 × 10^− 3^ mm²/s to 0.93 × 10^− 3^ mm²/s and 0.26 × 10^− 3^ mm²/s to 2.98 × 10^− 3^ mm²/s in the contralateral normal region. Although the normal region had higher values than the core, a substantial overall overlap of values was observed.

**Fig. 6:**
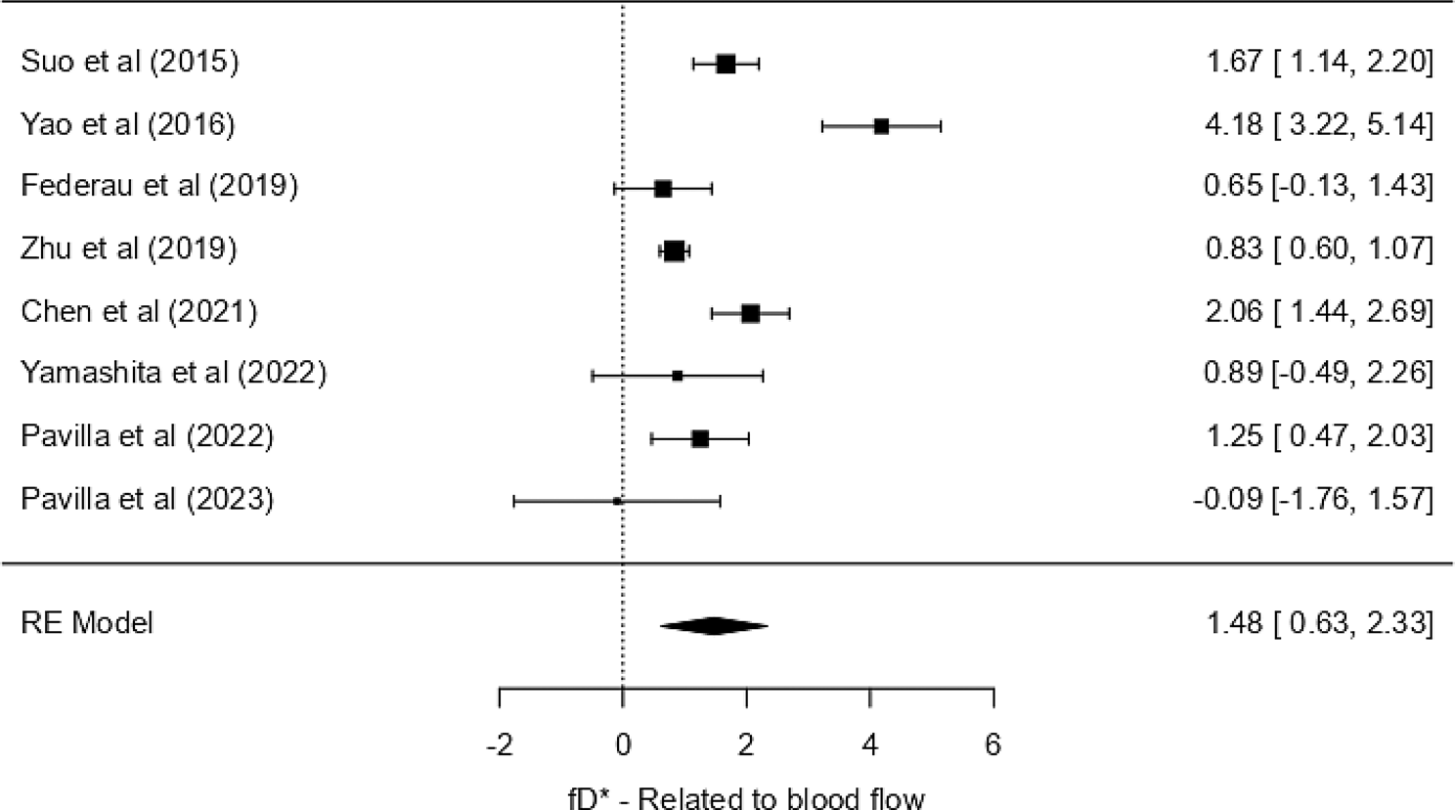
Forest plot for fD* parameter related to blood flow - sensitivity analysis

The weighted mean fD* in the core was 0.401 × 10^− 3^ mm²/s (95% CI: 0.368–0.433) and in the control it was 0.611 × 10^− 3^ mm²/s (95% CI: 0.571–0.652). Thus, a thorough quantitative distinction cannot be made. The random-effects model meta-analysis yielded a significant pooled SMD of 1.48 SD units (SE = 0.434, Z = 3.40, *p* < 0.001), with a CI of 0.63 to 2.33, indicating a large average difference in fD* between core and contralateral tissue. However, substantial heterogeneity was present among the studies (I² = 91.53%, tau² = 1.2913, Q = 60.965, *p* < 0.001), suggesting considerable variability in effect sizes across studies. The publication bias was not evident, as confirmed by non-significant Kendall’s Tau (*p* = 0.917) and Egger’s regression (*p* = 0.854) results, despite a large Fail-Safe N (419, *p* < 0.001). Due to disproportionately large values compared to other included studies, a sensitivity analysis was performed after excluding Zhu et al. [23]; However, the results remained consistent. Since the outlier with an exceptionally large confidence interval visually distorted the forest plot by compressing the confidence intervals of other studies around the line of no effect, the complete forest plot, including all studies, is provided in the supplementary material (Fig. S5b). The sensitivity analysis is demonstrated in Fig. 6 to enhance interpretability while ensuring methodological rigor and transparency in reporting. In the revised analysis, the standardized mean difference remained the same, 1.48 (95% CI: 0.633, 2.33), suggesting a moderate to large effect. However, several individual studies had CIs crossing zero, underscoring between-study variability. Despite a significant pooled effect, the high heterogeneity limits generalizability.

### Relationship between IVIM parameters and conventional diffusion and perfusion parameters

#### IVIM parameters and ADC values

Out of the 10 included studies, only two performed Pearson correlation analyses between mean values of ADC and IVIM parameters. Suo et al., in a dataset of 101 ischemic stroke patients, revealed a statistically significant positive correlation with D (*r* = 1, *p* < 0.001 in both core and control) and f (in core *r* = 0.541, *p* < 0.001; in control *r* = 0.262, *p* = 0.008). In contrast, no significant correlation was seen between ADC and IVIM parameters D* and fD* [17]. The other study showed a significant correlation between ADC and D (*r* = 0.599, *p* < 0.0001), suggesting that IVIM-D may reflect similar diffusion characteristics as ADC in acute ischemic stroke [30]. While preliminary evidence from the two studies directly comparing IVIM-D with ADC suggests a potential correlation, the limited number of studies precludes drawing a definitive conclusion.

#### IVIM parameters and ASL parameters

D* (fast ADC) showed a significant correlation with ASL CBF with *r* = 0.416 and *p* = 0.022 (where alpha = < 0.05) in the ischemic penumbral region. The reason stated by the authors for this correlation is that, since D* is inversely proportional to MTT and CBF is also inversely proportional to MTT, D* could have correlated significantly with CBF [20]. Another study showed fair to good positive correlation between fD* and ASL-CBF (*r* = 0.653, *p* < 0.001) and between f and ASL-CBF (*r* = 0.472, *p* = 0.012) [19], which was not seen in the previous study [20] The relationship with fD* could be explained by the fact that fD* is a product of f and D* and D* is closely related to CBF, while f is related to blood volume [9].

#### IVIM parameters and DSC parameters

Volumes of perfusion abnormality calculated on fD* and CBF showed the best agreement within ischemic volumes in the Bland-Altman analysis in one study that compared IVIM and DSC-PWI. Good agreement was seen between the f and CBV, with (95% values within the 95% CI). However, D* showed no correlation with MTT or Tmax [28].

## Discussion

This systematic review and meta-analysis put into perspective all the available findings that have assessed the IVIM technique in evaluating acute ischemic stroke. The first study to evaluate stroke using IVIM was performed by Wirestam et al. [24] in 1997. It evaluated the perfusion fraction f, which was reduced in the affected areas compared to the respective contralateral normal region. This finding is consistently seen in all the studies included in this review. Eventually, after over a decade, Federau at el [18]. showed the potential of using parameters other than perfusion fraction f. Further, Hu et al. [20] and Yao et al. [19] compared IVIM findings to ASL, emphasising the effectiveness of f and fD* parameters. One study pointed out the variation in values due to the collateral blood flow in the regions surrounding the core of the ischemic lesion, thus aiding in the delineation of the penumbra [27]. While ADC is a sensitive marker for detecting infarcted tissue and is sufficient to distinguish core from normal regions, it lacks specificity in identifying the ischemic penumbra. The IVIM model, by separating pure diffusion (D) from perfusion-related diffusion (D*, f), introduces a perfusion-sensitive dimension that may help identify tissue at risk but not yet infarcted. This is especially important in hyperacute settings where therapeutic decisions hinge on identifying salvageable tissue. Although our analysis focused on core versus control regions, future studies with voxel-level or ROI-based analysis of the penumbra may validate IVIM’s added value beyond what ADC alone can provide. Zhu et al. tested various combinations of b-values that would be effective enough to evaluate the minute changes in acute ischemic tissues and concluded that four b-values vis-à-vis 0, 50, 200, 1000 are sufficient and would reduce the scan time drastically, which is crucial in acute stroke, where immediate intervention is essential [23]. Chen et al. [29] reported a negative correlation between relative IVIM parameters (lesioned/contralateral parameter ratios) rADC (relative ADC) and rD (relative Diffusion) and clinical outcomes measured by baseline NIHSS, and a 90-day modified Rankin Scale (mRS) scale, which implies that lower baseline ADC and D values could lead to poorer long-term outcomes. Recently, two studies have been performed using a hybrid sequence that combines IVIM and DKI. This provides extraordinary information regarding the lesion considering non-gaussian information and a Kurtosis map [21, 30]. However, these studies were performed in small cohorts; Longitudinal studies with larger samples would be needed to validate the feasibility of such a hybrid sequence. Moreover, a contradicting result was shown in one of these studies concerning the D* and fD* parameters compared to the other included studies.

### Variability of parameters among the studies included for meta-analysis

#### D - true diffusion coefficient

D refers to the thermal diffusion of molecules across the tissue in a voxel, related to the conventional DWI/ADC parameters. The results indicate a strong and statistically significant SMD for the diffusion coefficient, but with substantial heterogeneity across studies. These differences could have occurred due to the substantial difference in the magnitude of the effect across studies calculated using the approximation method, as most studies did not report the effect size, 95% CI, or the p-value. It ranged from 1.75 × 10^− 3^ mm²/s [30] to 5 × 10^− 3^ mm²/s [27]. However, the latter study also showed a relatively low variance (0.0036), suggesting a more precise estimate than other studies. In contrast, the former study had a smaller sample size (*N* = 16) and a larger variance (0.04). It also used a hybrid IVIM-DKI sequence at 3T, which is a unique characteristic of this study that could be considered a methodological advancement over time. The limited number of studies comparing IVIM parameters with ADC restricts direct validation of IVIM-derived diffusion metrics. However, the observed strong correlations between ADC and D in both Suo et al. and Pavilla et al. support the potential of D as a surrogate for ADC in clinical stroke imaging, However, further validation is required. The absence of correlation between ADC and perfusion-related IVIM parameters (D*, fD*) further highlights the added physiological specificity offered by IVIM, particularly in capturing perfusion-independent diffusion characteristics. These findings reinforce the complementary role of IVIM alongside conventional ADC, though more studies are needed to validate this relationship in larger cohorts. Moreover, it also showed good correlation with 90-day functional outcome and might be able to predict long-term stroke outcomes in the acute setting.

#### f - perfusion fraction

The perfusion fraction is said to be homologous to CBV [31, 32]. It is known to have decreased in ischemic lesions [17]. Our findings are consistent with this fact, showing lower perfusion fraction values in the core compared to normal tissue, as the meta-analysis shows a statistically significant effect size. However, the perfusion fraction in the core is not completely zero, probably due to the Virchow-Robin Spaces (VRS) or perivascular spaces surrounding the perforating vessels that are known to enlarge in cases of inflammation or ischemic injury [33]. This parameter showed a positive correlation with ASL-CBF [19] and a good amount of agreement with DSC-CBV [23], too, thus providing perfusion-related information. However, these findings support the continued investigation of perfusion fraction as a biomarker in stroke management.

#### D* pseudo-diffusion coefficient

D* represents the motion of blood within the nutrient vessels, and not from typical thermal diffusion, and hence is referred to as the “pseudo-diffusion coefficient.” Theoretically, it is negatively correlated with the MTT [34]; however, no such correlation has yet been established in stroke studies. Our meta-analysis on D* demonstrated no significant pooled effect with effect sizes ranging from − 0.35 to 0.88. Notably, one study [21] reported a negative effect size, contrasting with the positive effect sizes observed in other individual studies. Furthermore, another study [23] reported exceptionally high mean D* values (core: 520.3 × 10^− 3^ mm²/s and control: 752.4 × 10^− 3^ mm²/s), leading to an extremely high variance (398,413.44). Such findings highlight the very low precision and wide confidence intervals, with considerable overlap between infarct and control regions. This variability likely stems from methodological and biological factors, including low SNR levels, partial volume effects, motion artifacts, and heterogeneity in patient cohorts (age, lesion location, and stroke severity). Importantly, the large uncertainty in the pooled estimate indicates that D* currently lacks clinical utility for differentiating viable from non-viable tissue. At best, it should be considered an exploratory parameter until more robust, standardized studies with age-matched cohorts and uniform NIHSS stratification are available.

#### fD* parameter related to blood flow

The significant effect size suggests that fD* is a potentially valuable parameter in clinical or imaging contexts, as it also showed a good correlation with ASL-CBF values in the studies analyzed. Since fD* reflects capillary perfusion and pseudo-diffusion, the wide CI crossing the zero mark may have stemmed from the large effect sizes of the studies, occurring perhaps due to the heterogeneity in collateral circulation, infarct progression, and individual patient hemodynamics. There could be many other probable explanations for the results we obtained. Yao et al. [19] reported the highest effect size of 4.18 with moderate variance (0.2401), suggesting great differentiation between core and control using fD* in their sample. This study had a prospective design, a moderate sample size (*n* = 38), participants were relatively younger (mean 55 years), and a larger number of b-values were used. Specific patient characteristics or study design factors might have driven the high effect size. In contrast, Pavilla et al. [30] reported a negative effect size with high variance, indicating a weak and possibly inverse relationship. However, this study had a small sample size (*n* = 15), which could have contributed to the higher variability and the negative effect estimate. The removal of a study [23] due to extreme values highlights the need for standardized protocols for image acquisition and measurement techniques to reduce variability. All these reasons highlight the complexity of interpreting fD* as a biomarker. While the overall effect remains statistically significant, the clinical relevance remains uncertain.

Collectively, the IVIM parameters (D, f, and fD*) demonstrate statistically significant differences between infarcted and contralateral tissue and correlate with established imaging metrics and functional outcomes. While these findings highlight their potential as biomarkers in stroke, the current evidence is insufficient to directly guide acute treatment decisions. Further prospective studies are needed to determine whether these IVIM-derived measurements could meaningfully inform clinical management or prognostication.

### Limitations of the study

This review yielded fewer articles; few studies could have been missed due to a stringent search strategy, and only 10 or fewer studies were included in the meta-analysis. However, these studies were published between 2015 and 2023, representing diverse geographical regions such as China, Switzerland, the USA, Japan, and France. Thus allowing generalizability to an extent. Multiple studies had a small sample size, which could have influenced our findings. Both retrospective and prospective study designs are represented in our study, but the retrospective studies that are typically prone to selective bias have larger sample sizes and may have driven the overall effect while overpowering the prospective study findings. The absence of consistent strategies to identify and adjust for confounders in several studies may have contributed to the heterogeneity in IVIM parameter estimates. These limitations highlight the need for more rigorously designed prospective studies with standardized protocols and robust adjustment for potential confounding factors. The overall study population included in the meta-analysis had patients with hyperacute, acute, or even subacute stroke patients; this could have influenced the range of values of the IVIM parameters. Finally, sources of heterogeneity were explored by performing sensitivity analyses excluding outlier studies with disproportionately large values (e.g., Zhu et al.), which confirmed the robustness of the pooled effect estimates. We also examined potential influences of scanner field strength and *b*-values, but found no consistent trends. However, due to the small number of studies and lack of uniform reporting of clinical covariates (age, NIHSS, lesion location), further subgroup or meta-regression analyses were not feasible.

### Scope for future research

Future studies should aim to use larger, more homogenous populations and standardized protocols in a multi-centric setting to better understand the potential role of the various parameters of IVIM in assessing stroke and its relationship with clinical outcomes. Studies should also systematically report acquisition parameters and clinical covariates to enable more robust moderator analyses. Advancements in hardware and software for improving the SNR and avoiding over- or underestimation of IVIM parameters using better fitting models that can suppress noise or partial volume effects should be considered to improve the overall estimation of values. Moreover, additional research should explore the longitudinal relationship between IVIM parameters and long-term stroke outcomes and their potential utility in tailoring individualized rehabilitation strategies. Machine learning or deep learning-based models that could fasten the diagnosis and give more accurate and precise output based on the IVIM parametric inputs could be explored and validated for routine use in acute ischemic stroke. Efforts are being made to introduce deep neural networks (DNNs) that rapidly generate IVIM parametric maps, thereby achieving superior fitting accuracy and reduced computation time compared to traditional fitting methods [35]. However, retraining may be required for different anatomical regions or optimised acquisition protocols specific for acute ischemic stroke. Such models could improve precision and speed in tissue segmentation, reducing interpretation time and human error, and could automatically differentiate infarct core from salvageable tissue, thereby supporting selection for reperfusion therapies. Furthermore, deep learning-assisted IVIM analysis may enhance outcome prediction, streamline workflow, and provide actionable insights for acute stroke triage and personalized patient care [36, 37].

## Conclusion

This review concludes that D, f, and fD* significantly distinguish between the core and control regions. Additional studies would be required to comment on D*. Nonetheless, this cannot diminish the potential of the IVIM sequence in providing valuable insights into the tissue microstructure, diffusion, perfusion, and ischemia non-invasively within a short acquisition time without the need for a contrast agent. It can provide voxel-based perfusion estimation, not influenced by the surrounding large vessels, which happens in techniques like DSC and ASL, thus providing more sensitive information than is available in these conventional techniques. It shows great promise in neuroimaging and effective diagnosis of acute ischemic stroke with a multifaceted approach.

## Supplementary Information

Below is the link to the electronic supplementary material.

Supplementary Material 1

Supplementary Material 2

Supplementary Material 3

## Data Availability

All data generated or analysed during this study are included in this published article [and its supplementary information files].

## Abbreviations

ADC: Apparent Diffusion Coefficient
ASL: Arterial Spin Labelling
CBF: Cerebral Blood Flow
CBV: Cerebral Blood Volume
DSC: Dynamic Susceptibility Contrast
IVIM: Intravoxel incoherent motion
NIHSS: National Institute of Health Stroke Severity
MRI: Magnetic Resonance Imaging
MTT: Mean Transit Time
ROI: region of interest
