# Supplementary material for: Quantitative estimation of intravoxel incoherent motion parameters in acute ischemic stroke: A Systematic review and meta-analysis
Source: BMC Med Imaging. 2025 Nov 12;25:462. doi: 10.1186/s12880-025-01997-3 (PMC12613901; doi:10.1186/s12880-025-01997-3)
Supplement: Supplementary file 1 — Supplementary Material 1 [file 12880_2025_1997_MOESM1_ESM.pdf]

Supplementary Material S3

Table S3: **Intravoxel Incoherent Motion Parametric values for core and control obtained from the studies included in the meta-analysis**

**Study label**	**Sample Size**	**D core** ($\times 10^{-3}$ mm^2/s) **Mean±SD**	**D control** ($\times 10^{-3}$ mm^2/s) **Mean±SD**	**f Core** (%) **Mean±SD**	**f Control** (%) **Mean±SD**	**D* Core** ($\times 10^{-3}$ mm^2/s) **Mean±SD**	**D* Control** ($\times 10^{-3}$ mm^2/s) **Mean±SD**	**fD* core** ($\times 10^{-3}$ mm^2/s) **Mean±SD**	**fD* control** ($\times 10^{-3}$ mm^2/s) **Mean±SD**
Federau et al (2014)[1]	17	0.39±0.08	0.75±0.09	2.6±1.9	5.6±2.5	NR	NR	NR	NR
Suo et al (2015)[2]	101	0.42±0.1	0.72±0.07	4.29±2.01	7.97±2.03	10.2±4.17	10.87±4.75	0.49±0.27	0.94±0.42
Yao et al (2016)[3]	38	0.47±0.1	0.75±0.08	2.85±0.99	7.91±1.82	32.94±11.44	37.71±6.38	0.93±0.49	2.98±0.82
Federau et al (2019)[4]	34	0.5±0.06	0.8±0.1	4.6±3.3	6.3±2.2	9.1±5.9	10.9±8.1	0.48±0.4	0.74±0.74
Zhu et al (2019)[5]	20	0.54±0.11	0.84±0.1	4.45±2.62	7.22±2.93	33.18±6.4	35.75±4.61	0.16±0.12	0.26±0.12
Zhu et al (2019)[6]	58	0.56±0.15	0.82±0.107	16.63±9.34	22.19±6.53	520.3±631.2	752.4±545.8	10000±16820	17710±14590
Chen et al (2021)[7]	39	0.28±0.08	0.56±0.1	4.42±1.69	5.7±1.24	14.65±8.45	22.09±7.94	0.62±0.32	1.28±0.51
Yamashita et al (2022)[8]	29	0.45±0.09	0.76±0.11	3.22±2.93	5.55±2.16	22.6±7.24	24.3±4.94	0.728±0.702	1.35±0.59

Pavilla et al (2022)[9]	5	0.48±0.09	0.83±0.1	4±2	6±2	17.6±17	11.6±15.1	0.5±0.4	1±1.5
Pavilla et al (2023)[10]	15	0.58±0.2	0.93±0.01	2.5±2.7	4.3±3.8	NR	NR	0.6±0.85	0.52±0.53

D = True diffusion Coefficient, f = Perfusion fraction, D = Pseudo-diffusion coefficient, fD* = Blood flow related, SD = Standard Deviation, NR = Not Reported*

References

1. Federau C, Sumer S, Becce F, Maeder P, O'Brien K, Meuli R, et al. Intravoxel incoherent motion perfusion imaging in acute stroke: initial clinical experience. Neuroradiology. 2014;56:629–35.

2. Suo S, Cao M, Zhu W, Li L, Li J, Shen F, et al. Stroke assessment with intravoxel incoherent motion diffusion-weighted MRI. NMR Biomed. 2016;29:320–8.

3. Yao Y, Zhang S, Tang X, Zhang S, Shi J, Zhu W, et al. Intravoxel incoherent motion diffusion-weighted imaging in stroke patients: initial clinical experience. Clin Radiol. 2016;71:938.e11-938.e16.

4. Federau C, Wintermark M, Christensen S, Mlynash M, Marcellus DG, Zhu G, et al. Collateral blood flow measurement with intravoxel incoherent motion perfusion imaging in hyperacute brain stroke. Neurology. 2019;92.

5. Zhu G, Federau C, Wintermark M, Chen H, Marcellus DG, Martin BW, et al. Comparison of MRI IVIM and MR perfusion imaging in acute ischemic stroke due to large vessel occlusion. International Journal of Stroke. 2020;15:332–42.

6. Zhu G, Heit JJ, Martin BW, Marcellus DG, Federau C, Wintermark M. Optimized Combination of b-values for IVIM Perfusion Imaging in Acute Ischemic Stroke Patients. Clin Neuroradiol. 2020;30:535–44.

7. Chen F, Dai Z, Yao L, Dong C, Shi H, Dou W, et al. Association of cerebral microvascular perfusion and diffusion dynamics detected by intravoxel incoherent motion-diffusion weighted imaging with initial neurological function and clinical outcome in acute ischemic stroke. PeerJ. 2021;9:e12196.

8. Yamashita K, Kamei R, Sugimori H, Kuwashiro T, Tokunaga S, Kawamata K, et al. Interobserver Reliability on Intravoxel Incoherent Motion Imaging in Patients with Acute Ischemic Stroke. American Journal of Neuroradiology. 2022;43:696–700.

9. Pavilla A, Gambarota G, Arrigo A, Saint-Jalmes H, Mejdoubi M. Toward an Intravoxel Incoherent Motion 2-in-1 Magnetic Resonance Imaging Sequence for Ischemic Stroke Diagnosis? An Initial Clinical Experience With 1.5T Magnetic Resonance. J Comput Assist Tomogr. 2022;46:110–5.

10. Pavilla A, Gambarota G, Signaté A, Arrigo A, Saint-Jalmes H, Mejdoubi M. Intravoxel incoherent motion and diffusion kurtosis imaging at 3T MRI: Application to ischemic stroke. Magn Reson Imaging. 2023;99:73–80.
