# Supplementary material for: Quantitative estimation of intravoxel incoherent motion parameters in acute ischemic stroke: A Systematic review and meta-analysis
Source: BMC Med Imaging. 2025 Nov 12;25:462. doi: 10.1186/s12880-025-01997-3 (PMC12613901; doi:10.1186/s12880-025-01997-3)
Supplement: Supplementary file 2 — Supplementary Material 2 [file 12880_2025_1997_MOESM2_ESM.pdf]

Supplementary Material S4

Table S4: Imaging-related data extracted from the studies included for meta-analysis.

Author	Year	SO-IT (Mean±SD)	MRI field Strength	No. of B Values	B-values used	MRI Machine	IVIM Acquisition Parameters
C. Federau	2014	45.5±40.3	3	16	0, 10, 20, 40, 80, 110, 140, 170, 200, 300, 400, 500, 600, 700, 800, 900	Seimens Trio, Verio, Skyra	ST: 4mm; FOV: 270x270; Matrix: 225 x 225; TR: 4000; TE: min.; Acc Factor: 2; FS; Scan Time: 3.7 min
Shiteng Suo	2015	78±39.6	3	9	0, 20, 50, 100, 150, 200, 500, 800 and 1000	HDxt GE MRI	TR: 6000; TE: 88; FOV: 260 x 260; Matrix size: 192 x 192; 18 slices; ST: 5mm; Spacing: 1.5; acc factor: 2; Scan time: 5 min
Y. Yao	2016	156±66	3	15	0, 20, 40, 80, 110, 140, 170, 200, 300, 400, 500, 600, 700, 800, 900, 1000	GE Discovery 750	ST:5 mm; FOV: 240x240 mm2; Matrix Size:160x160; TR: 5200ms; TE: minimum; Scan time: 4.51 min
C. Federau	2019	6±2.95	3	6	0, 50, 100, 150, 200, 1000	NR	TR: 4200; TE: 78; FOV: 249 x 240; ST: 5mm; Matrix: 128 x 128; Acc. Factor: 2; Scan time: 1.11 min.
Guangming Zhu	2019	8.3±5.07	3	6	0, 50, 100, 150, 200, 1000	GE MR750	TR: 4500; TE: 77; FOV: 240 x 240; Matrix: 128 x 128; ST: 5mm; Scan time: 2min.
Guangming Zhu	2019	9.3±11.7	3	6	0, 50, 100, 150, 200, 1000	GE MR750	TR: 4500; TE: 77; FOV: 240 x 240; Matrix: 128 x 128; ST: 5mm; Scan time: 2min.
Fei Chen	2021	29.5±12.59	3	15	0, 10, 20, 40, 80, 110, 140, 170, 200, 300, 400, 500, 600, 800, 1000	HDxt GE MRI	TR = 5,825 ms, TE = 94 ms, FOV = 24 × 24 cm2 , slice thickness/gap = 5 mm/1.5 mm, matrix size = 128 × 128, acceleration factor = 2; Scan time: 7.11 min.
K. Yamashita	2022	26±61.85	1.5	6	0, 50, 100, 150, 200, and 1000	Achieva, Phillips	TR: 3500 ms; TE: 75 ms; flip angle: 90°; NEX: 1; Sections=23; ST= 5 mm; interslice gap=1.5 mm; FOV, 220x220 mm; matrix= 124x105; and scan time= 1.24 min
Auda Pavilla	2022	4.6±5.5	1.5	8	0, 60, 80, 300, 400, 900, 1000, and 1500	Optima MR 450 GE	TR: 3000; TE: 75; ST: 4mm; FOV: 250 x 250; Matrix: 128 x 128; Scan Time: 4.38 min
Auda Pavilla	2023	6±7.5	3	9	0, 30, 60, 80, 300, 400, 900, 1000 and 1500	MR750W SIGNA, GE	TR: 6500; TE: 80; ST: 4mm; FOV = 240 × 240; matrix: 128 × 128
