# Supplementary material for: Quantitative estimation of intravoxel incoherent motion parameters in acute ischemic stroke: A Systematic review and meta-analysis
Source: BMC Med Imaging. 2025 Nov 12;25:462. doi: 10.1186/s12880-025-01997-3 (PMC12613901; doi:10.1186/s12880-025-01997-3)

Supplementary material S5

Fig. S5a - Forest plot for D* (including all the studies)

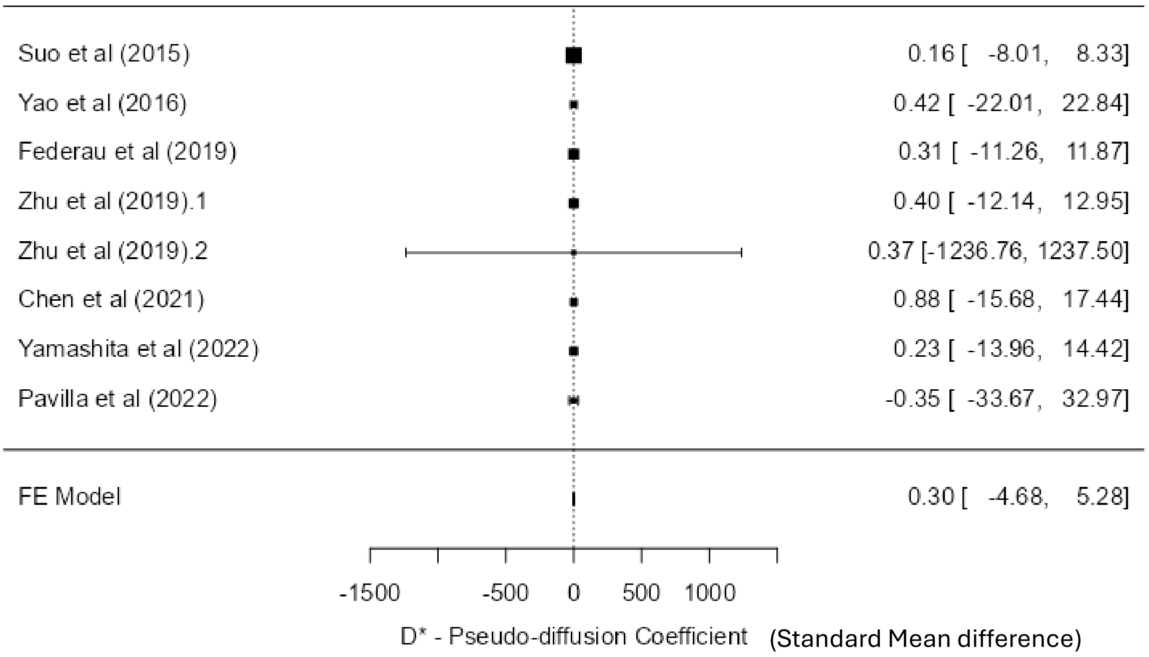

Figure 1:S1 - Forest plot for D (including all the studies)*

Fig S5b: Forest plot for fD* (including all the studies)

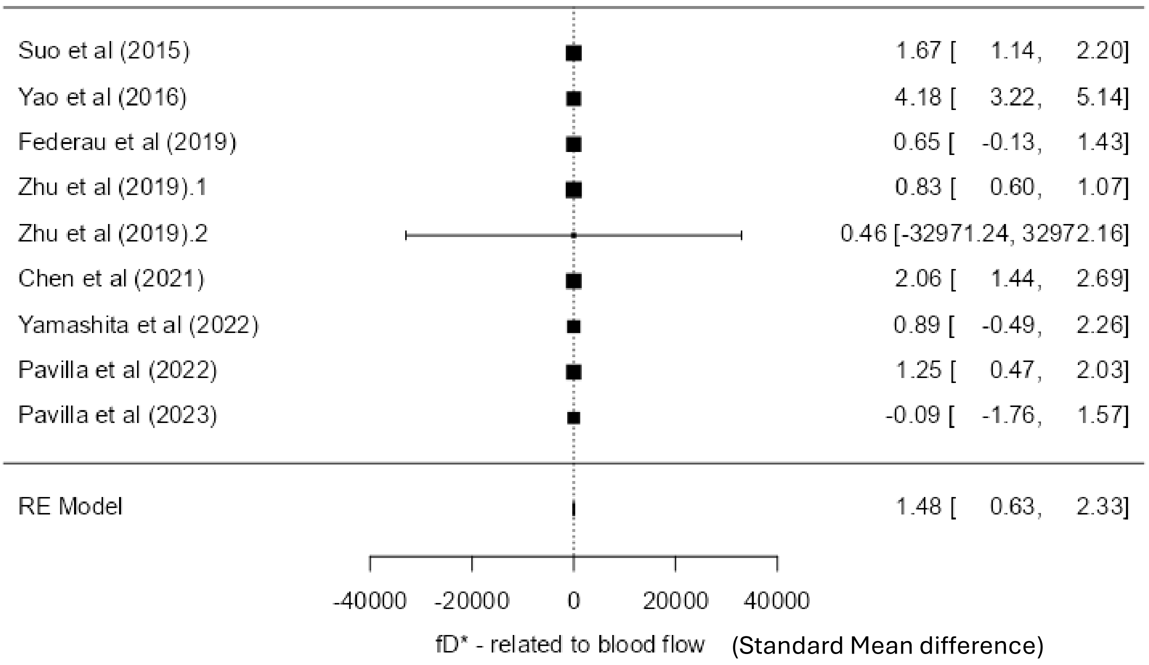

Supplement: Supplementary file 3 — Supplementary Material 3 [file 12880_2025_1997_MOESM3_ESM.pdf]
